# Evaluating plant immunity using mass spectrometry-based metabolomics workflows

**DOI:** 10.3389/fpls.2014.00291

**Published:** 2014-06-24

**Authors:** Adam L. Heuberger, Faith M. Robison, Sarah Marie A. Lyons, Corey D. Broeckling, Jessica E. Prenni

**Affiliations:** ^1^Proteomics and Metabolomics Facility, Colorado State UniversityFort Collins, CO, USA; ^2^Department of Soil and Crop Sciences, Colorado State UniversityFort Collins, CO, USA; ^3^Department of Horticulture and Landscape Architecture, Colorado State UniversityFort Collins, CO, USA; ^4^Department of Biochemistry and Molecular Biology, Colorado State UniversityFort Collins, CO, USA

**Keywords:** metabolomics, plant defense, plant pathogen, LC-MS, GC-MS

## Abstract

Metabolic processes in plants are key components of physiological and biochemical disease resistance. Metabolomics, the analysis of a broad range of small molecule compounds in a biological system, has been used to provide a systems-wide overview of plant metabolism associated with defense responses. Plant immunity has been examined using multiple metabolomics workflows that vary in methods of detection, annotation, and interpretation, and the choice of workflow can significantly impact the conclusions inferred from a metabolomics investigation. The broad range of metabolites involved in plant defense often requires multiple chemical detection platforms and implementation of a non-targeted approach. A review of the current literature reveals a wide range of workflows that are currently used in plant metabolomics, and new methods for analyzing and reporting mass spectrometry (MS) data can improve the ability to translate investigative findings among different plant-pathogen systems.

## Introduction

The advent of the three central “omics” platforms: genomics, transcriptomics, and proteomics has been invaluable to systems biology (Fiehn et al., 2001; Ge et al., 2003). While often described as a hypothesis-generating science, an omics approach allows for the ability to elucidate complex phenotypes at the systems-level. For investigations in plant immunity, such studies have been instrumental in discovering fundamental interactions between genes, transcripts, proteins, and metabolites that define plant defense phenotypes (Maleck et al., 2000; Rajjou et al., 2006).

Primary and secondary metabolites are known to be critical to the plant immune response, and the addition of a metabolomics workflow to the omics toolbox is essential for systems-level research. Plant metabolomics is a relatively new plant omics technique (Fiehn et al., 2000a) but it has not seen widespread use due to major challenges in chemical detection and data analysis. For example, while the chemical structure of genes, transcripts and proteins is well defined, there is a wide variation in structure and abundance of small molecules in plants (Dixon, 2001; Gershenzon and Dudareva, 2007). This diversity presents a major analytical challenge for global metabolomics analysis.

Here, we review metabolomics studies that evaluate plant immunity with an emphasis on mass spectrometry (MS) in its many forms. A metabolomics workflow encompasses procedures for metabolite extraction, detection by MS, data analysis, and biological interpretation. The differences between targeted and non-targeted workflows are also discussed. A review of the current literature revealed that a wide range of workflows are currently used in plant metabolomics and illustrates that the addition of a metabolomics workflow to the omics toolbox can provide new (and validate existing) hypotheses in plant immunity.

## A large and diverse group of small molecules mediate the response to biotic stress

**Figure 1:**
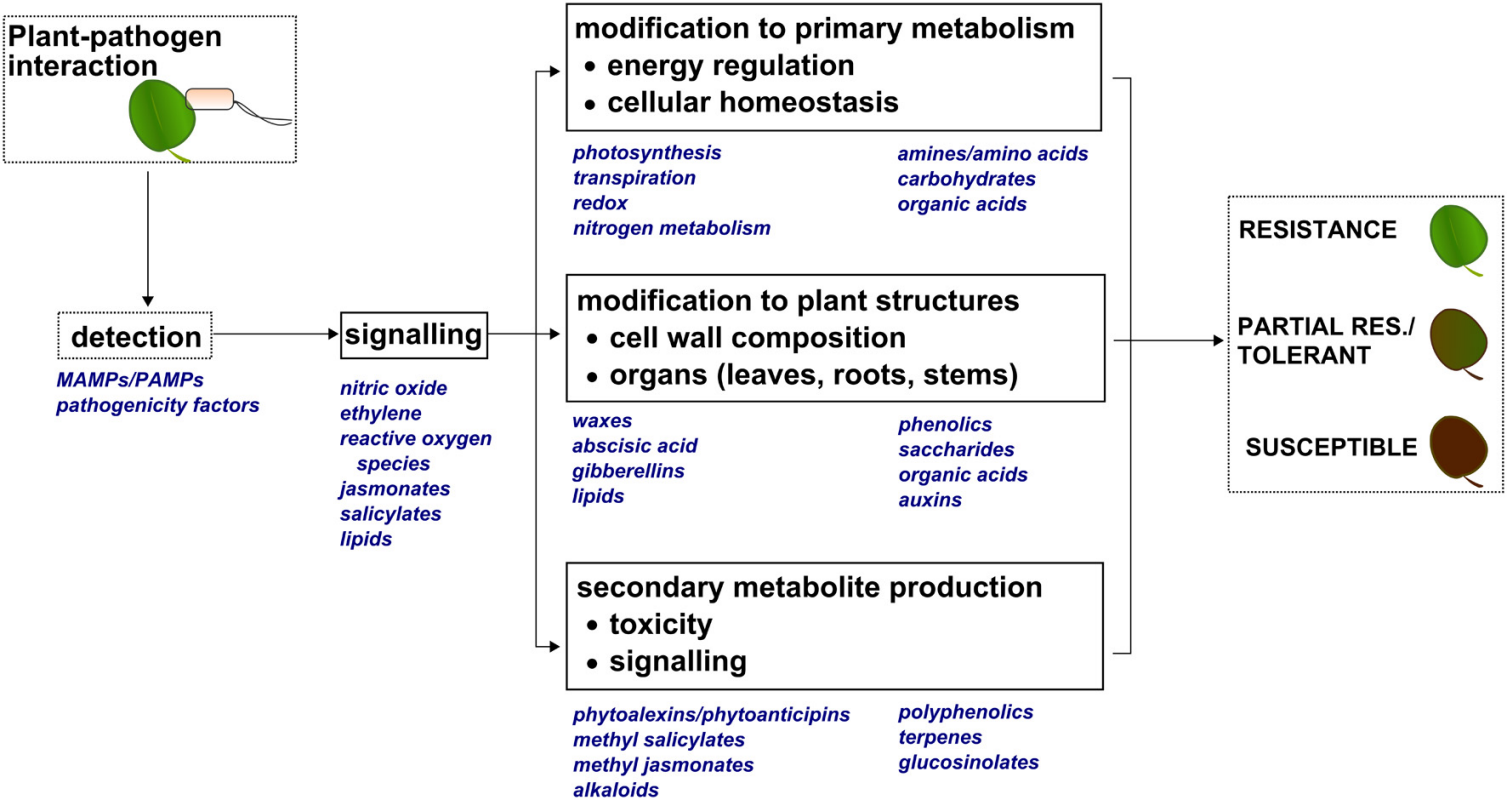
**Molecular and physiological modifications that can occur during a plant-pathogen interaction**. Upon detecting Pathogen- or Microbe-Associated Molecular Patterns (PAMPs, MAMPs), small molecules are produced that act as molecular signals to induce changes in primary metabolism, modify plant structures, and produce secondary metabolites. These events are also driven by small molecules, and ultimately define the resistant, partially resistant/tolerant, or susceptible plant phenotype.

Plant metabolic factors are major contributors to plant defense (Pare and Tumlinson, 1999; Dixon, 2001; Bolton, 2009). Figure 1 provides an illustration showing plant metabolite classes and physiological processes known to be related to plant defense. Upon detection of Pathogen- or Microbe-Associated Molecular Patterns (PAMPs, MAMPs), a molecular signaling cascade results in physiological modifications (Berger et al., 2007), and this may ultimately define the resistant, tolerant, or susceptible phenotype. Like other global omics workflows (e.g., transcriptomics and proteomics), metabolomics provides the ability to globally survey the small molecule components involved in detection, signaling, and physiological, morphological, and chemical responses resulting from pathogen infection.

Cell signaling represents the first molecular event associated with plant immunity. Signaling occurs very early in the plant molecular response to infection and can be observed as an increase in abundance of specific non-volatile (e.g., salicylate) or volatile (e.g., ethylene) molecules in the initial hours after pathogen infection. Volatile compounds such as nitric oxide, ethylene, methyl jasmonate, and methyl salicylate are key mediators of systemic acquired resistance (Ecker and Davis, 1987; Gundlach et al., 1992; Delledonne et al., 1998; Park et al., 2007). Many variants of these hormones have also been described for their role in immunity, such as the conjugate of jasmonate and isoleucine (Staswick and Tiryaki, 2004). In addition to direct effects such as pathogen toxicity and cell wall fortification (Bradley et al., 1992; Lamb and Dixon, 1997), reactive oxygen species such as superoxide and hydrogen peroxide can also act as non-volatile signals of pathogen infection (Torres et al., 2006). Many non-hormone plant compounds have dual metabolic and signaling effects such as the amino acids homoserine and asparagine (Yang et al., 2005) and sphingolipids (Ng et al., 2001; Vicente et al., 2013). Additionally, the interaction among signaling compounds also affects immunity, for example cross-talk between ethylene and jasmonate (Lorenzo et al., 2003), or nitric oxide and jasmonate (Wang and Wu, 2005).

Along with cell signaling molecules, primary metabolic compounds are also involved in the plant defense response. Primary metabolites related to defense are comprised of a diverse class of molecules that include carbohydrates, organic acids, amines/amino acids, and lipids reviewed in Rojas et al. (2014). In response to a pathogen, a shift in primary metabolic compounds can be associated with changes in energy metabolism (sucrose; Scharte et al., 2005), nitrogen metabolism, (amino acids; Tavernier et al., 2007), and cellular homeostasis such as pH and redox status (malate, ascorbate, tocopherol; Sakano, 2001; Foyer and Noctor, 2005; Roos et al., 2006; Liu et al., 2010). A shift in primary metabolism may support a series of physiological and morphological modifications to inhibit pathogen colonization or growth as primary metabolites have been shown to be associated with structural modifications. For example, changes to carbohydrates and phenolic organic acids have been associated with cell wall modifications related to insects and fungi (Barros-Rios et al., 2011; Cao et al., 2011). Other non-hormonal metabolites mediate defense-related processes such as stomatal closure (malate; Dittrich and Raschke, 1977), leaf rolling (malate, citrate; Saglam et al., 2010), and callose deposition (uridine diphosphate-glucose; Schlupmann et al., 1993).

A diverse group of secondary metabolites are also responsible for mediating plant defense. Phytoanticipins (basally produced) and phytoalexins (induced upon pathogen infection) can have a direct toxic effect on pathogens. These secondary metabolites are often observed as various terpenoids, phenolics, and other miscellaneous nitrogen or sulfur containing compounds such as indoles, alkaloids, and glucosinolates (Dixon, 2001). Furthermore, there is extensive diversity within each of these chemical classes, often relating to specialized functions such as toxicity or volatile signaling (Gershenzon and Dudareva, 2007).

Overall, the plant defense response is largely a product of the interaction of a diverse class of metabolites. Thus, chemical profiling experiments should be designed to maximize the coverage of chemical classes to simultaneously assay for events related to both cell signaling and primary and secondary metabolism. Metabolomics, the global analysis of a broad class of small molecule compounds, is therefore an important tool enabling investigations of the molecular basis of plant immunity in plant-pathogen interaction systems.

## Mass spectrometry workflows to survey small molecules associated with plant immunity

### Overview

In a standard metabolomics workflow, metabolites are assayed by extracting compounds from plant tissue in a solvent, and subsequently detecting and quantifying compounds using various chemical detection platforms (e.g., MS, nuclear magnetic resonance, ultraviolet absorbance). MS-based metabolite detection is a powerful tool for investigations of plant metabolism due to its sensitivity for low-abundant molecules and flexibility for the detection of multiple chemical molecular classes. Therefore, MS is well-suited to investigate the signaling, physiological, and other chemical events associated with plant defense. However, while many compounds related to plant defense are well-defined, there is currently no metabolite extraction protocol or MS detection platform that can survey the entire defense-related metabolome in a single experiment. Metabolite extraction procedures are inherently biased toward solubility of the molecule in the choice of solvent, specifically for differences in polarity or pH. Furthermore, MS detection platforms are biased in their compatibility of a particular molecule with a mode of ionization or detection. For example, electrospray ionization (ESI) MS can differentially apply positive or negative charges to molecules, and certain compounds vary in their propensity to form positively charged and negatively charged ions.

The ability to globally profile highly complex mixtures of plant extracts is enhanced by coupling chromatography with MS detection. Thus, a “metabolomics platform” refers to the combination of chromatography and MS. The two most commonly utilized metabolomics platforms include liquid chromatography-mass spectrometry (LC-MS) and gas chromatography-mass spectrometry (GC-MS) (Kopka et al., 2004).

**Figure 2:**
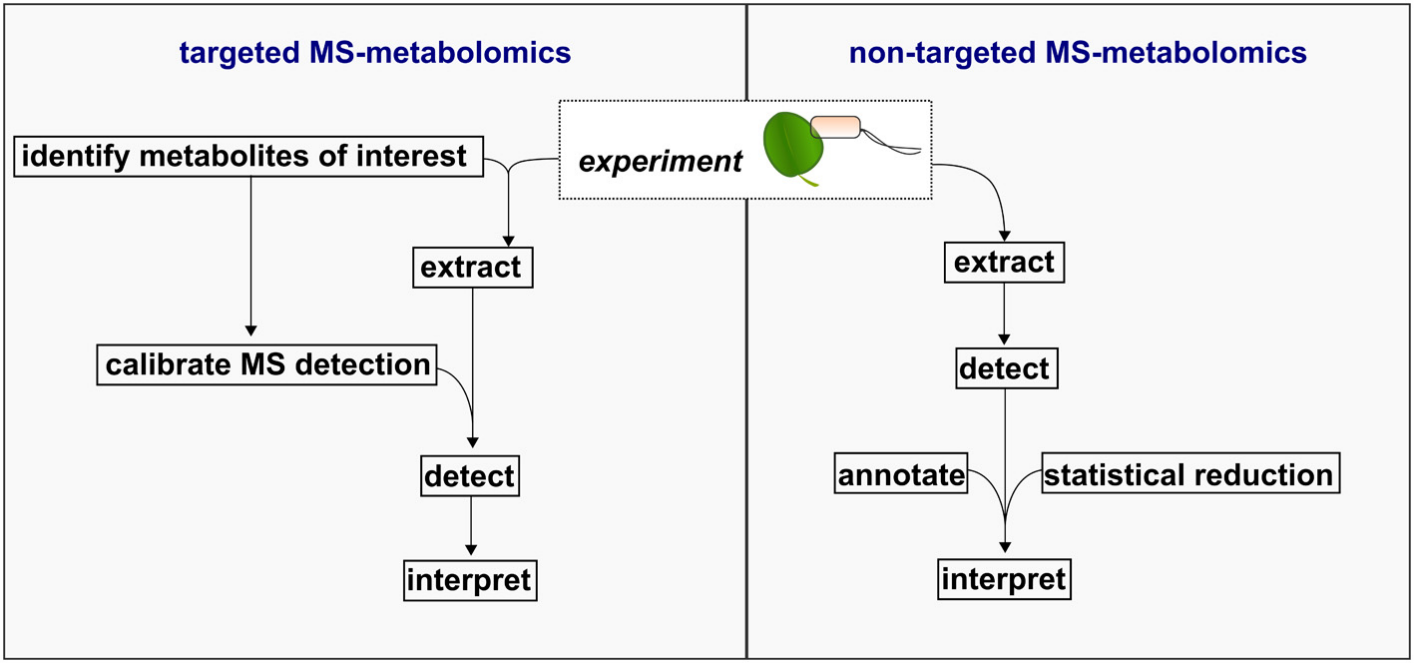
**Schematic of targeted and non-targeted MS-metabolomics workflows**.

Following data acquisition and processing, MS-metabolomics data is often expressed as a matrix of molecular features defined by (i) chromatography elution time, (ii) mass (mass/charge ratio) within the mass error of the instrument, and (iii) intensity of the mass signal as a quantitative unit. While GC- and LC-MS platforms provide superior sensitivity in detecting a diverse set of compounds, annotating the detected molecular feature as a metabolite is the major bottleneck in MS-metabolomics workflows (Wishart, 2011), and is further complicated due to the extensive diversity in plant compounds. The annotation bottleneck has resulted in the delineation between two metabolomics workflows: targeted and non-targeted, the latter also known as “untargeted,” “unbiased,” or “global” metabolomics (Figure 2). Here, the general procedure associated with each workflow is reviewed in the context of metabolomics investigations related to plant defense.

### The targeted MS-metabolomics workflow

A targeted metabolomics workflow is designed for the analysis of a subset of specific metabolites in a single experiment, thus requiring an *a priori* knowledge of the compounds of interest. The classes and types of metabolites involved in plant defense are well-defined, and therefore the targeted MS-metabolomics workflow has major utility in plant-pathogen interaction experiments. An example hypothesis amenable to a targeted workflow may be: a pathogen of interest is expected to alter amino acid and monosaccharide metabolism after 24 h of infection, but differentially affect a wildtype and mutant plant.

There are multiple ways in which a workflow can be targeted to specific metabolites. First, a metabolite extraction procedure can be developed to maximize recovery of the target compounds from the plant tissue of interest. This improves the overall sensitivity of detecting the compounds of interest, for example by extracting in a specific pH, polarity, or through the use of solid phase extraction enrichment. For example, an extraction solvent of 50% methanol (methanol/water) would extract metabolites in a single phase, whereas an experiment targeting amino acids and carbohydrates may extract in chloroform/methanol/water and only assay the aqueous fraction of the biphasic system. The latter extraction procedure could also provide improved sensitivity in a targeted lipid MS-profiling experiment due to the reduced complexity of the sample. If two compounds chromatographically co-elute, the mass spectrometer will have to split the available acquisition time across multiple compounds, resulting in diminished sensitivity overall. Thus, a targeted reduction in sample complexity can result in significant increases in sensitivity for compounds of interest.

In a targeted experiment, the mass spectrometer is specifically tuned to detect a specific set of compounds. Typically, authentic standards (if available) are first run to define the time and mass parameters associated with each compound. This targeted acquisition approach addresses the metabolite identification bottleneck by only collecting data on metabolites with known masses and retention times. Furthermore, running authentic standards prior to a complex sample can provide an overview of which metabolites can and cannot be detected using the chosen extraction solvent and metabolomics platform. For LC-ESI-MS, certain metabolites may be only detected with positive or negative ionization, and many metabolites may not be retained using a particular chromatographic method. GC-MS metabolomics is dependent on transitioning the metabolites of interest to a gas phase, and multiple derivatization procedures are often employed to increase volatility required for GC, including methoximation critical for carbohydrate analysis by GC-MS (Schweer, 1982), trimethylsilylation (TMS), or the TMS-alternative tertbutyldimethylsilylation that is compatible with amino acids but not high molecular weight carbohydrates (Fiehn et al., 2000b; Lisec et al., 2006). For example, based on the example hypothesis presented above, a methoximation-TMS/GC-MS procedure would be likely amenable to many (but not all) of the amino acids and carbohydrates of interest.

A targeted metabolomic workflow requires relatively simple data analysis procedures. Data normalization can be conducted using stable isotope labeled internal standards for each compound of interest, or for compound classes if not all labeled standards are available. For example, if glucose is a specific target in the plant extract, then glucose-^13^C_6_ can be added to the extraction solvent to account for analytical variation (e.g., extraction or derivatization efficiency, column degradation, detector source contamination). The internal standard is collected as a separate, single data point, and analysis software can be directed to automatically normalize to the standard before the data is exported for downstream interpretation. In addition, while the MS detector results in data at the “molecular feature” level, retention times and masses are pre-determined using authentic standards, thus the data can be automatically output as “metabolites” instead of “m/z at a given time.” For MS-metabolomics, this data reduction can improve the statistical robustness of the experiment as fewer tests result in decreased false discovery (Broadhurst and Kell, 2006), and minimizes the potential to over-fit multivariate models by reducing the presence of analytical artifacts in the data matrix (described in section Non-targeted MS-metabolomics Workflows).

While a targeted workflow can improve in the overall sensitivity and specificity of an experiment, there are also limitations due to the significant time, labor, and cost of developing a targeted MS method and the requirement for authentic standards. Additionally, chromatographic retention times can be unstable over periods of time and across instruments and laboratories, for example due manual solvent mixture preparation (LC) or chromatography column variation and/or degradation (LC or GC). Thus, the time parameter of a detection method needs to be calibrated, internally or externally, across experiments and laboratories. Furthermore, a targeted method is dependent on *a priori* knowledge, which is inherently biased toward a current understanding of which molecules are important to plant defense in the system under study. Thus, a targeted workflow will not provide information on previously undescribed molecular events within a specific plant-pathogen interaction.

### Non-targeted MS-metabolomics workflows

A non-targeted metabolomics workflow is designed to globally profile all detectable metabolites in a single experiment. It requires little to no *a priori* knowledge of which metabolites would be involved in plant immunity. Often, a non-targeted approach has the ability to detect many (if not all) of the same metabolites included in a targeted assay, depending on obstacles related to instrument sensitivity and sample complexity, but has the added advantage of collecting novel information. An example hypothesis amenable to a non-targeted workflow may be: a pathogen of interest is expected to alter plant secondary metabolism after 24 h of infection, but differentially within a wildtype and mutant plant. In contrast to the targeted workflow, “secondary metabolism” provides little focus for metabolite extraction and detection techniques, as secondary metabolites are incompletely characterized for most species, and the targeted approach would fail to detect many of the important compounds involved in this response.

While metabolite extraction and detection are inherently biased, the procedures can be designed to maximize coverage of the metabolome to included broad detection of amines/amino acids, organic acids, lipids, alkaloids, and many other compound classes. Non-targeted metabolite extractions can be conducted using miscible organic/aqueous solvents (e.g., single phase mixtures such as 80% methanol or isopropanol/acetonitrile/water). Internal standards may be added during this extraction step, and/or the data can be normalized using computational procedures during data processing (e.g., total signal or quantile normalization). A variety of normalization procedures have been developed (Sysi-Aho et al., 2007; Veselkov et al., 2011; De Livera et al., 2012), and while the normalization procedure is a critical step in the non-targeted workflow, there is little consensus as to which normalization procedures are the most robust. In general, a procedure that accounts for biological (plant), technical (extraction), analytical (detection), and computational (MS peak detection and alignment) variability should be employed.

In addition to metabolite extraction and data normalization, non-targeted MS-metabolomics is highly dependent on chemo-informatic procedures to assign metabolite identifications from MS data. The initial steps (mass peak detection, grouping, alignment, deconvolution) can be performed using various computational platforms (e.g., Smith et al., 2006; Lommen, 2009; Xia et al., 2009) or other MS vendor-specific software. The resulting data is often a matrix of molecular features defined by mass (mass/charge ratio), retention time, and a quantitative value relative to the normalization procedure. The entire data matrix is often interrogated using uni- or multi-variate statistical analyses (e.g., ANOVA, PCA, PLS, correlation) to identify which molecular features varied within the experimental design. However, a major flaw in conducting statistical analyses on the resulting data set is assumed independence among the variables (molecular features) in the data. MS requires a compound to be ionized prior to detection, a process that often results in multiple mass signals that correspond to the same molecule. For LC-ESI-MS, these often include the presence of isotopes (^12^C, ^13^C) adducts (Na^+^, K^+^), charge states ([M+H]^+^, [M+2H]^2+^), and in-source fragments/neutral losses ([M-glycosyl group]). GC-MS metabolomics methods mostly use electron impact ionization, which is a high-energy procedure that results in highly fragmented molecules and include many (~5–100) mass signals that correspond to a single metabolite. In contrast to a targeted workflow, where data will be acquired for only one or few of the mass signals that correspond to a metabolite, all detectable mass signals will be acquired in a non-targeted MS-metabolomics experiment and processed as an independent variable in the overall dataset. Computational procedures to cluster this redundant data have been developed for LC-MS (Tautenhahn et al., 2007; Broeckling et al., 2012; Kuhl et al., 2012) and GC-MS (Stein, 1999).

The next major step in the non-targeted workflow is the annotation of molecular features as metabolites. The annotation procedure is somewhat hierarchical in structure, with the first and most confident methods based on the matching of mass spectral and retention time data to in-house or external metabolite databases of authentic standards. Similar to a targeted metabolomics experiment, an in-house database requires the acquisition and curation of mass spectra and retention times. While ideal, a large in-house spectral library requires significant resources to develop due to the time and cost to purchase or acquire authentic standards, and acquire and curate the MS data. Furthermore, many plant authentic standards are not available and many compounds are species- or situation-specific, such as phytoalexins that are only produced upon the detection of a pathogen. Therefore, it is critical to develop a metabolite annotation procedure that is independent of any *a priori* knowledge about the mass spectra or retention time of pathogen-related compounds.

**Figure 3:**
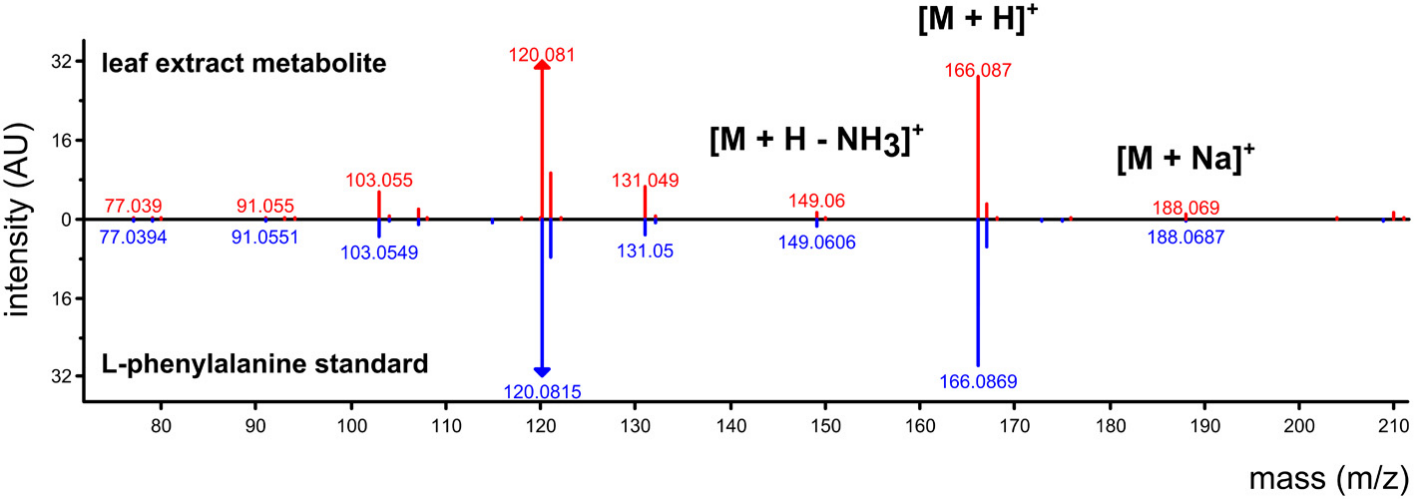
**Annotation of an LC-ESI-MS detected (positive ionization) metabolite using a metabolite database for non-targeted MS acquisition**. In a non-targeted workflow, each mass signal is treated as an independent variable for statistical analysis, but the entire spectrum informs on the metabolite identity. In a targeted workflow, L-phenylalanine standard would be pre-run to identify 166.087 m/z as the major mass signal to monitor during a pre-determined time window.

For GC-MS and LC-ESI-MS metabolomics, mass spectra can be searched against external resources such as NIST standard reference (www.nist.gov), Massbank (Horai et al., 2010), Metlin (Smith et al., 2005), and Golm (Kopka et al., 2005) spectral databases. If the external databases lack a perfect match, partial matches may indicate a similar molecular structure to the unknown compound in the experiment. In addition to spectral matching, individual masses within a spectrum can be manually interpreted to inform on the structure of the precursor molecule. A manual interpretation usually requires high-resolution mass information (i.e., less error in measuring mass), and may depend on identifying patterns in the data to identify the molecular weight of the target compound. For example, LC-ESI-MS data run in positive ionization mode may result in the detection of [M+H]^+^, [M+Na]^+^, and [M+H]^+^ – H_2_O in a single spectrum that correspond to [M+1.008], [M+22.990], and [M+1.008 – 18.015]. For example, Figure 3 shows a spectrum that was obtained from plant leaf extracts using non-targeted LC-ESI-MS profiling in positive mode followed by data processing using the algorithm described in Broeckling et al. (2012). The top panel shows the experimental spectrum matched against the spectrum from an authentic standard of L-phenylalanine. It is important to note that if data reduction via clustering was not performed on the data, each of the mass signals in this spectrum would have been considered an independent data point even though they are derived from the same metabolite.

In the absence of confident identification by spectral matching or spectral interpretation, a molecular weight-based search is the next-best alternative. Individual mass values can be searched in external chemical databases, however this approach is largely dependent on both the mass error of the instrumentation and the manual interpretation of the experimental mass spectrum to identify a putative molecular weight. *In silico* fragmentation tools such as MetFrag (Wolf et al., 2010) can provide additional confidence in a putative match, as well as elemental composition analysis available in various software. If possible, the putative metabolite annotations should be validated with a comparison to an authentic standard. If a standard is unavailable, the evidence for an annotation should be included as supplementary data in a published manuscript (Sumner et al., 2007). Due to the requirement for manual interpretation of mass spectra, this annotation process is a major bottleneck in the time and cost of non-targeted metabolomics experiments.

In summary, there are distinct differences in the underlying assumptions and procedures within targeted or non-targeted MS-metabolomics workflows. A targeted workflow requires significant effort prior to the experiment and only results only in the detection of a pre-determined set of metabolites. However, targeted workflows benefit from less complex data analysis procedures, are more robust to statistical assumptions, and offer improved sensitivity due to optimized extraction and detection procedures. Alternatively, non-targeted metabolomics workflows allows for novel discoveries in a broader range of compounds, including unknown compounds and metabolites unique to a specific plant species. The non-targeted MS-metabolomics workflows allow for more simple extraction and detection procedures, however result in highly complex data with increased false discovery burden and interdependence of features (variables) requiring significantly more effort in data analysis and interpretation.

## Example studies that utilize MS-metabolomics workflows in plant immunity

### Overview

**Figure 4:**
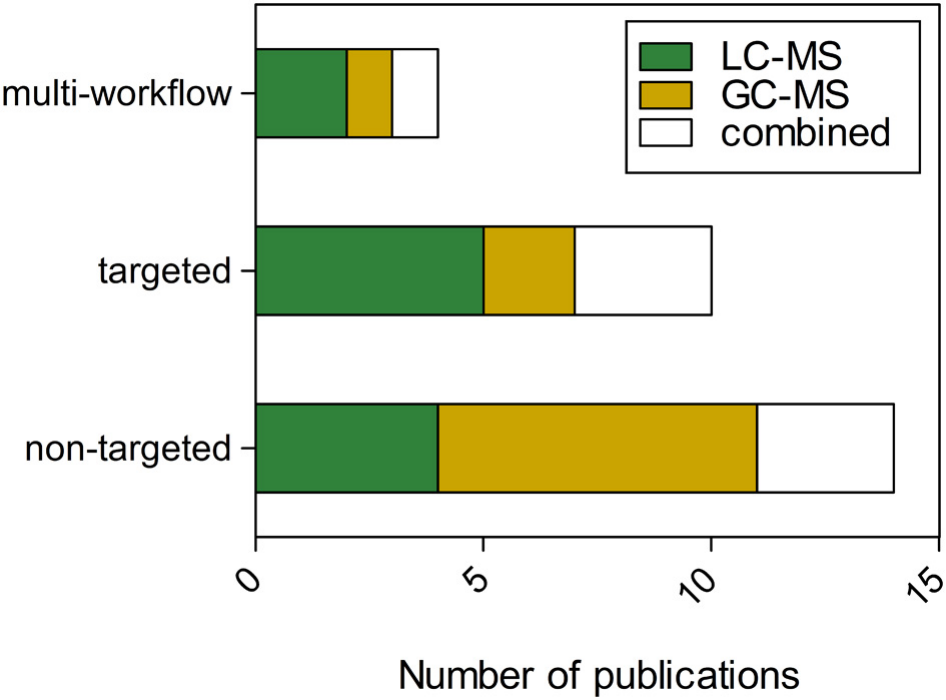
**Distribution of publications across workflows and platforms for MS-metabolomics related to plant immunity**. “Combined” refers to studies that utilized LC and GC platforms. “Multi-workflow” refers to a publication that utilized both a targeted and non-targeted workflow.

A collection of investigations was identified that utilized MS-metabolomics workflows to help answer key questions in plant immunity. The search terms “plant,” “pathogen,” and “metabolomics” were included as keywords in Web of Science and resulted in a total of 70 peer reviewed publications. A random subset of these publications (28 in total) was further examined to determine the distribution of plant species, pathogen classes, and types of metabolites observed using MS-metabolomics (Table 1). The publications revealed a distribution across bacteria, fungi, oomycete, insects, epiphytes, and viruses, and showed several common metabolite classes found to be related to disease resistance. The publication set also illustrated that, to date, LC-MS platforms were more frequently employed for targeted workflows and GC-MS for non-targeted workflows. (Figure 4). A portion of the publications performed a combination of targeted and non-targeted metabolomics (“multi-workflow”), utilizing elements of both workflows to optimize the experiment. For further review, 16 of the 28 publications that corresponded to plant-fungal interactions were interrogated to determine major similarities and differences in analytical platforms and detected metabolites among the targeted and non-targeted MS-metabolomics workflows.

**Table 1:** **Subset of MS-metabolomic studies with workflows, platforms, and example plant metabolites associated with disease**.

**Pathogen**	**Plant**	**Workflow**	**Platform**	**Example metabolites**	**References**
Bacteria	*Arabidopsis*	Non-targeted	GC-MS	Salicylate, azelaic acid	Jung et al., 2009
	*Arabidopsis*	Non-targeted	LC-MS	Camalexin	Beets et al., 2012
	*Arabidopsis*	Targeted	GC-MS	Salicylates, jasmonates, camalexin	Mishina and Zeier, 2007
	*Arabidopsis*	Targeted	LC-MS	Salicylates, glucosinolates, camalexin, auxins, amino acids	Truman et al., 2010
	*Arabidopsis*	Targeted	LC-MS, GC-MS	Glycerol, glycerol-3-phosphate, salicylates, jasmonates, azelaic acid, lipids	Chanda et al., 2011
	*Arabidopsis*	Multi-workflow	GC-MS	Organic acids	O'brien et al., 2012
	*Citrus*	Non-targeted	GC-MS	Amino acids, organic acids, sugars	Cevallos-Cevallos et al., 2012
	*Nicotiana benthamiana*	Non-targeted	LC-MS	No annotation performed	Lee et al., 2013
	*Nicotiana benthamiana*	Non-targeted	GC-MS	Butyl 2-pyrrolidone-5-carboxylate	Park et al., 2007
Fungi	*Arabidopsis*	Non-targeted	GC MS	Amino acids, organic acids, sugars	Botanga et al., 2012
	*Arabidopsis*	Targeted	LC-MS	Indole-3-carboxylic acid	Gamir et al., 2012
	*Brachypodium distachyon*	Targeted	LC-MS	Lipids	Allwood et al., 2006
	*Citrus unshiu*	Non-targeted	GC-MS, LC-MS	Amino acids, organic acids, sugars, lipids	Yun et al., 2013
	*Eucalyptus globulus*	Non-targeted	GC-MS	Volatiles	Hantao et al., 2013
	*Helianthus annuus*	Non-targeted	GC-MS	Amino acids, organic acids, sugars, chlorgenic acid	Peluffo et al., 2010
	*Hordeum vulgare*	Non-targeted	LC-MS	Phenylpropanoids	Bollina et al., 2011
	*Lupinus angustifolius*	Semi-targeted	GC-MS, LC-MS	Flavonoids	Wojakowska et al., 2013
	*Nicotiana tabacum*	Non-targeted	GC-MS, LC-MS	Terpenoids, coumarins, jasmonates, salicylates	Tugizimana et al., 2014
	*Nicotiana tabacum*	Multi-workflow	LC-MS	Phenylpropanoids	Madala et al., 2013
	*Oryza sativa*	Targeted	LC-MS	Diterpenoid phytoalexins, hydroxycinnamaldehydes	Kishi-Kaboshi et al., 2010
	*Solanum tuberosum*	Non-targeted	GC-MS, FT-ICR-MS	Amino acids, organic acids, sugars, lipids, alkaloids	Aliferis and Jabaji, 2012
Oomycetes	*Nicotiana benthamiana, Solanum tuberosum*	Targeted	GC-MS	Oxylipins	Saubeau et al., 2013
	*Nicotiana tabacum*	Non-targeted	LC-MS	Phenylpropanoid-polyamine conjugates, amines, oxylipins	Cho et al., 2013
	*Vitis vinifera*	Targeted	LC-MS	Stilbenes	Malacarne et al., 2011
	*Zingiber zerumbe*	Non-targeted	GC-MS	Organic acids, phenolics, terpenes	Keerthi et al., 2014
Viruses, epiphytes, pests	*Gracilaria chilensis*	Non-targeted	LC-MS	Oxylipins, jasmonates	Weinberger et al., 2011
	*Nicotiana tabacum*	Targeted	GC-MS	Capsidiol	Matros et al., 2006
	*Oryza sativa*	Targeted	GC-MS	Methyl salicylate, methyl benzoate volatiles	Zhao et al., 2010

### Example targeted MS-metabolomics studies of plant-fungi interactions

The number of studies that relied on targeted MS-metabolomics workflows of fungi was limited. One investigation used ESI-MS to target lipids and hormones predicted to be involved in the interaction between *Brachypodium distachyon* and *Magnaporthe grisea* (Allwood et al., 2006). Several lipids and hormones were predicted to be important based on previous characterization using non-MS chemical detection techniques. As expected, the authors describe variation in phospholipids to be the major plant response phenotype. Wojakowska et al. used a combination of targeted and non-targeted extraction and detection workflows to characterize the metabolic response of lupin (*Lupinus angustifolius*) associated with the fungus *Colletotrichum lupini* and/or its toxins (Wojakowska et al., 2013). The study describes a targeted extraction procedure to evaluate changes in cuticle metabolites (e.g., waxes) combined with non-targeted GC-MS, and a separate extraction and detection procedure for LC-MS detection of flavonoids. The study described major changes in both aglycone and conjugates of isoflavones, and the variation occurred at an earlier time-point and with greater intensity with *C. lupine* extracts than the pathogen itself.

Several additional targeted MS-metabolites studies characterized the metabolic effect of specific defense elicitors or stress metabolites. A study in rice (*Oryza sativa*) reported that metabolic changes occurred when plants were exposed to a chitin elicitor, and used LC-MS/MS (tandem MS) to detect the elicitor-induced production of several phytoalexins (Kishi-Kaboshi et al., 2010). Similarly, Gamir et al. used a targeted LC-MS/MS method to evaluate the effect of β-aminobutyric acid, a priming agent that induces callose deposition in *Arabidopsis*, and found major effects on hormone metabolism (Gamir et al., 2012). In this study, the authors describe an approach for detecting both the basic moieties and conjugated forms of hormones using a targeted workflow. For example, many auxins are variants of an indole moiety (116 m/z), and it is difficult to scan for all auxins using targeted metabolite profiling. Therefore, the reported targeted MS workflow involved (i) scanning for m/z values that correspond to the 116 m/z indole moiety and (ii) validating the full structure of the conjugation form using an orthogonal LC-ESI-MS experiment. They found that β-aminobutyric acid influenced the 116 m/z ion from plant extracts, and confirmed that this ion corresponded to an indole-3-carboxylic acid after validation with a commercial authentic standard.

### Example non-targeted MS-metabolomics studies of fungi

MS-metabolomics can be used to investigate the general plant response without an emphasis on disease resistance. Aliferis et al. reported the use of multiple MS detection platforms to describe the general metabolic response of potato sprouts (*Solanum tuberosum*) to *Rhizoctonia solani* (Aliferis and Jabaji, 2012). They reported shifts in primary metabolism that agree with general theories of nutrient remobilization, specifically a decrease in carbohydrates and amino acids upon infection. They observed both an increase and decrease in select organic acids and lipids, and an overall increase in potato alkaloids. An *Arabidopsis-Alternaria brassicicola* interaction study used non-targeted GC-MS and described 128 and 249 molecular features that varied after 9 and 24 h of infection including several sugars, organic acids, and amino acids (Botanga et al., 2012).

Metabolomics studies are also relevant for plant systems as a means to elucidate the mechanisms underlying genetic sources of resistance. Sunflower (*Helianthus annuus*) resistance to the necrotrophic fungal pathogen *Sclerotinia sclerotiorum* is associated with at least 44 quantitative trait loci (reviewed in Ronicke et al., 2005). Peluffo et al. characterized sunflower resistance to the necrotrophic pathogen *Sclerotinia sclerotiorum* using non-targeted GC-MS and found 63 metabolites including sugars (trehalose), organic acids (glycerate, citrate, succinate), amino acids (asparagine, valine, tyrosine) and secondary metabolites (chlorogenic acid) to be associated with a tolerant phenotype (Peluffo et al., 2010). Similar to *S. sclerotiorum* infection, an MS-metabolomics study of *Fusarium graminearum* revealed a modulation in amino and organic acids (Bollina et al., 2010) in barley (*Hordeum vulgare*). Using a non-targeted LC-MS approach they found 496 molecular features that varied among resistant and susceptible lines, 50 of which could be annotated using a non-targeted informatics workflow and included organic acids, amino acids, phenylpropanoids, flavonoids, fatty acids, and terpenoids. Interestingly, *F. graminearum* is known to produce deoxynivalenol (DON), a terpene virulence factor, but this compound was not detected. However, because a non-targeted workflow was employed the glycosylated form of DON was detected, indicating an active detoxification event that had not previously been described in barley. A comparable non-targeted LC-MS metabolomics study was conducted with *F. graminearum* and *Triticum aestivum*, and the authors reported 473 (rachis) and 340 (spikelet) molecular features that differed between the resistant and susceptible lines due to the *Fhb1* locus (Gunnaiah et al., 2012). Furthermore, the study reported that DON levels did not vary between the resistance and susceptible lines, indicating a different mechanism of resistance than in barley. In contrast, the authors noted drastic changes in phenylpropanoids (including related enzymes) that supports lignification as a key mediator of resistance.

To analyze fungal elicitors of plant defense, Madala et al. utilized non-targeted LC-MS metabolomics and showed that *N. tabacum c*ells exposed to the stress-metabolite isonitrosoacetophenone induced variation in phenylpropanoid and flavonoid metabolism (Madala et al., 2013). A similar study characterized the effect of ergosterol on *N. tabacum c*ells using non-targeted GC- and LC-MS (and other non-MS detection platforms), and found major metabolomic variation after 18 h of incubation with 300 nM ergosterol (Tugizimana et al., 2014). The major metabolic shifts occurred in secondary metabolites (terpenoids, coumarins, lignin precursors) and hormonal signaling molecules (jasmonate, salicylate-glucoside). In addition, heat treatment, a non-chemical elicitor of defense against post-harvest infection in citrus fruits, was investigated for its protection against fungal infection. A non-targeted LC-MS and GC-MS metabolomics study showed that organic acids (succinate), amines (ornithine), phenylpropanoids (flavonoids and lignin), and hormones (jasmonate) all increased in the pericarp of *Citrus unshiu* (mandarin), providing new insights into the molecular basis of heat-treatment induced resistance to fungal infection (Yun et al., 2013).

Another study identified volatile metabolites associated with the interaction between *Eucalyptus globulus* and the necrotrophic fungal pathogen *Teratosphaeria nubilosa* using non-targeted GC-MS (Hantao et al., 2013). More than 40 compounds were identified that can be used as biomarkers of disease, potentially before visual or non-volatile chemical traits can be observed. A similar volatile biomarker-discovery study was described for *Citrus sinensis* infection with *Candidatus liberibacter* (Aksenov et al., 2014), highlighting the potential use of chemical biomarkers of disease with non-destructive sampling during the growing season.

Taken together, these non-targeted MS-metabolomics studies consistently showed that the regulation of primary and secondary metabolites is important for resistance in plant-fungal interactions. While the targeted MS-metabolomics workflows described metabolic changes associated with fungal infection, the non-targeted studies provided a much broader view of metabolism. Thus, even though metabolite identification was a clear bottleneck in many of the non-targeted studies, each investigation allowed for an improved understanding of the plant response to fungal infection.

## Conclusion

MS is a high-throughput and sensitive chemical detection platform that can be used to globally characterize the metabolome and elucidate the molecular mechanisms that govern plant-pathogen interactions. MS-metabolomics is becoming an important tool to characterize the metabolic basis of resistance to pathogens in non-model systems represented in the extensive chemical diversity of the plant kingdom. The two major MS-metabolomics workflows, targeted and non-targeted, can provide important information that is complimentary to more traditional genomics approaches. The targeted MS-metabolomics workflow has the advantages of simple data analysis procedures and improved statistical robustness, however it is dependent on *a priori* knowledge of which metabolites are important for plant defense. The targeted workflow also requires significant up-front labor and costs to calibrate the MS acquisition method for the metabolites of interest. In contrast, the non-targeted metabolomics workflow provides the ability to sample a broad range of chemical classes, and thus plant metabolism, in a single experiment. However, it is limited by bottlenecks in metabolite identification and false assumptions in traditional statistical analyses. While still considered a relatively new field, the annotation bottlenecks in non-targeted metabolite profiling are predicted to significantly improve as the MS metabolite spectral databases mature. In addition, sharing of unknown spectra as supplemental data upon publication or to a public resource such as Massbank (Horai et al., 2010) can improve the general understanding of which chemicals, albeit unknown, are consistently associated with plant immunity. It is therefore recommended that mass spectra, which are the information collected in an MS-experiment, should be included in publications of non-targeted metabolomics experiments.

### Conflict of interest statement

The authors declare that the research was conducted in the absence of any commercial or financial relationships that could be construed as a potential conflict of interest.

## Abbreviations

MS: mass spectrometry
UPLC: ultra performance liquid chromatrography
GC: gas chromatography
ESI: electrospray ionization
PCA: principal component analysis
PLS: partial least squares.
